# Supplementary material for: Complexity analysis of heartbeat-related signals in brain MRI time series as a potential biomarker for ageing and cognitive performance
Source: Eur Phys J Spec Top. 2022 Oct 21;232(1):123–33. doi: 10.1140/epjs/s11734-022-00696-2 (PMC9988766; doi:10.1140/epjs/s11734-022-00696-2)
Supplement: Supplementary file 1 — (pdf 1568 KB) [file 11734_2022_696_MOESM1_ESM.pdf]

Supplementary Material:

Complexity analysis of heartbeat-related signals in Brain MRI time series as a potential biomarker for ageing and cognitive performance

David López Pérez[1,2], Arun L.W. Bokde[3] & Christian Kerskens[2]

[1]Institute of Psychology, Polish Academy of Sciences, Warsaw, Poland

[2]Institute of Neuroscience, Trinity College, Dublin, Ireland

[3]Trinity College Institute of Neuroscience and Cognitive Systems Group, Discipline of Psychiatry, School of Medicine, Trinity College Dublin, Ireland

Supplementary Figures

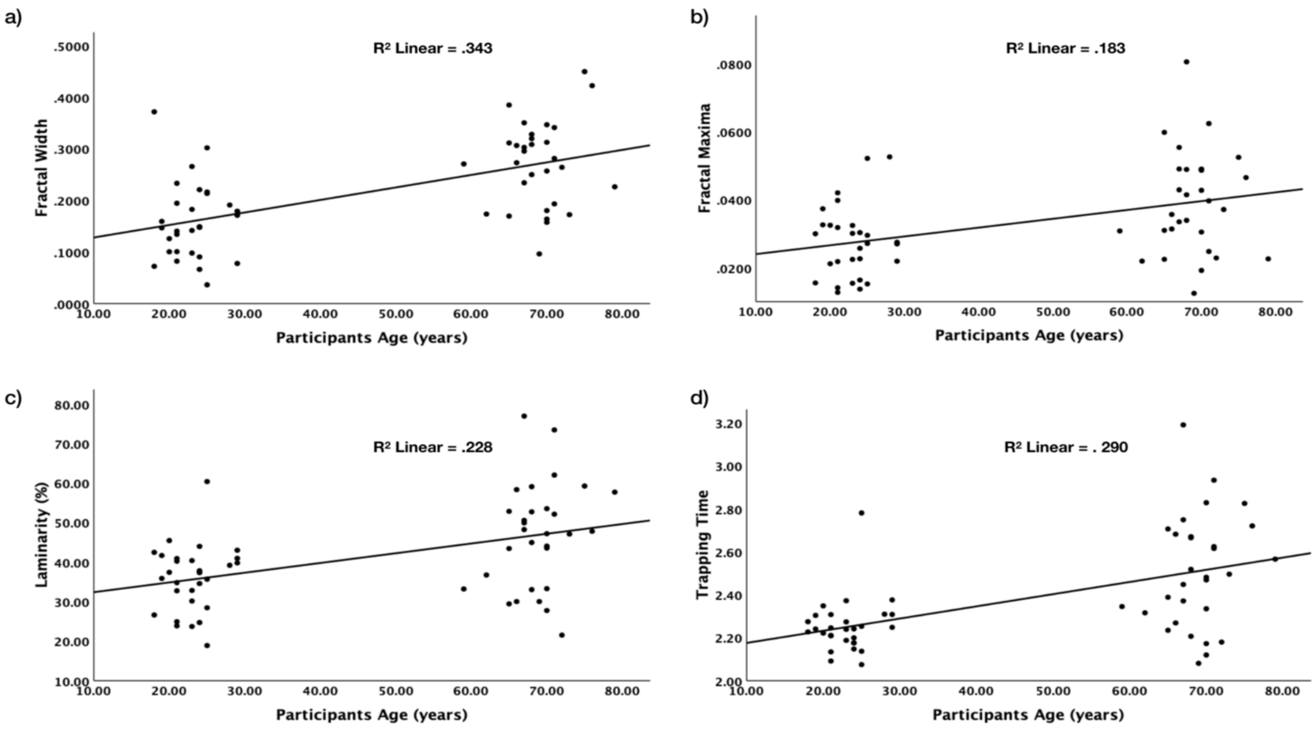

Supplementary Figure 1. *Linear regressions between (a) fractal width, (b) fractal maxima, (c) laminarity and (d) trapping time, and participants age.*

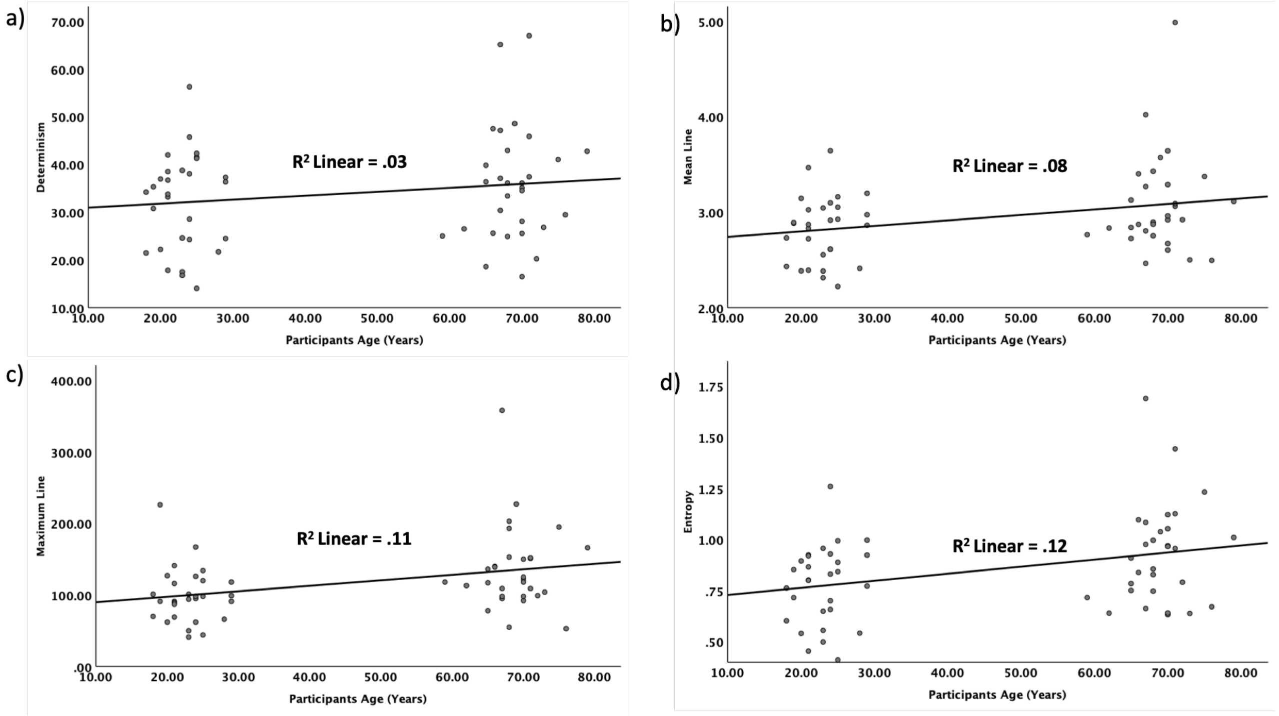

Supplementary Figure 2. Linear regressions between (a) determinism, (b) mean line, (c) maximum line and (d) entropy, and participants age.

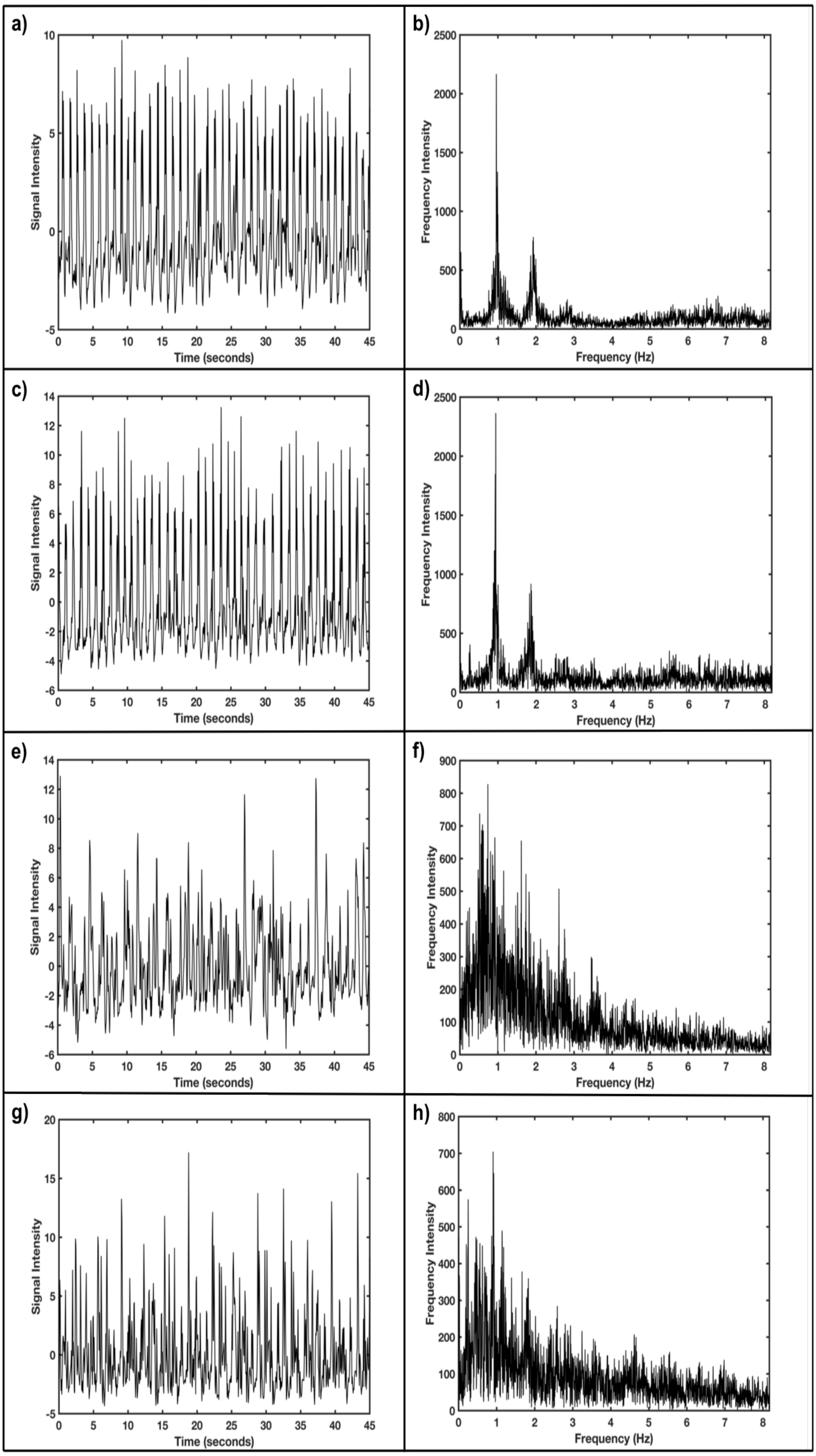

Supplementary Figure 3. *Example of the time-series as well as its frequency spectra for two young (a-c and b-d) and two healthy old adults (e-g and f-h). These examples are representing the typical results in these groups.*

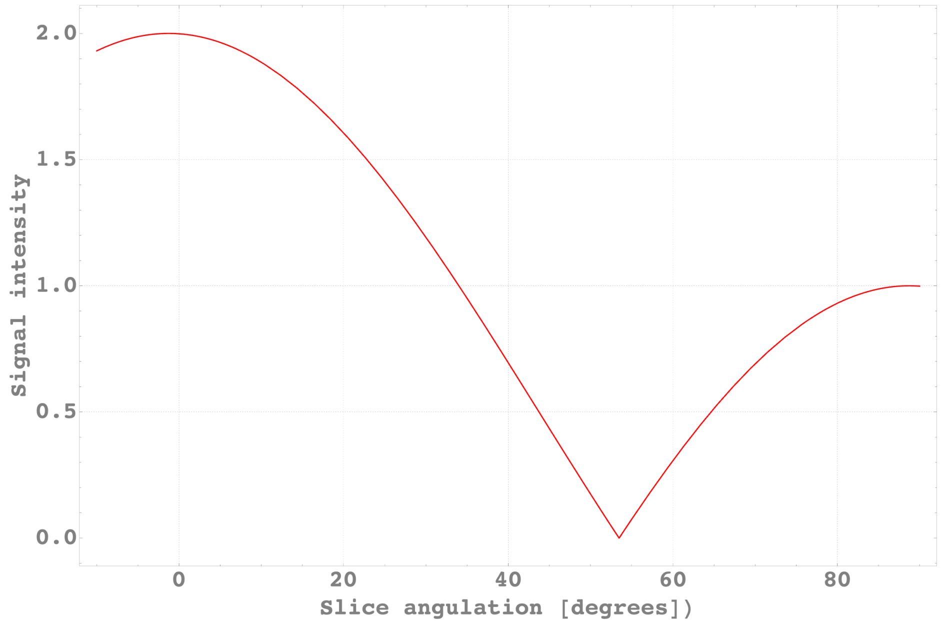

Supplementary Figure 4. *Signal amplitude depending on the slice orientation. A minimum value is observed at the magic angle (aprox. 55 degrees).*

Supplementary Analysis

Multiple Logistic Regression for the RQA parameters

The logistic regression model with all the RQA variables in only survive the first step with TT being enough to classify both age groups.

Variables in the Equation

		B	S.E.	Wald	df	Sig.	Exp(B)
Step 1[a]	Zscore(TT)	2.405	.708	11.546	1	<.001	11.075
	Constant	.480	.372	1.666	1	.197	1.616

a. Variable(s) entered on step 1: Zscore(TT).

With the variables not added into the model because no model improvement was found when adding them.

Variables not in the Equation

			Score	df	Sig.
Step 1	Variables	Zscore(Det)	.144	1	.704
		Zscore(MaxLine)	2.401	1	.121
		Zscore(MeanLine)	3.141	1	.076
		Zscore(Ent)	2.724	1	.099
		Zscore(Lam)	.299	1	.584
	Overall Statistics		14.082	5	.015

The final classification table shows that both age groups can be classified with an accuracy of 78%

Classification Table[a]

			Predicted		
			Age		Percentage Correct
Observed			.00	1.00	
Step 1	Age	.00	25	4	86.2
		1.00	9	21	70.0
	Overall Percentage				78.0

a. The cut value is .500

Multiple Logistic Regression for the Fractals

The logistic regression model with all the Fractals variables in only survive the first step with W being enough to classify both age groups.

Variables in the Equation

		B	S.E.	Wald	df	Sig.	Exp(B)
Step 1[a]	Zscore(fractalWidth)	1.768	.462	14.653	1	<.001	5.859
	Constant	.188	.330	.325	1	.569	1.207

a. Variable(s) entered on step 1: Zscore(fractalWidth).

With the fractal maxima not added into the model because no model improvement was found when adding them.

Variables not in the Equation

			Score	df	Sig.
Step 1	Variables	Zscore(fractalMax)	.465	1	.496
	Overall Statistics		.465	1	.496

The final classification table shows that both age groups can be classified with an accuracy of 74.5%

Classification Table[a]

Observed			Predicted Age .00	Predicted Age 1.00	Percentage Correct
Step 1	Age	.00	22	7	75.9
		1.00	8	22	73.3
	Overall Percentage				74.6

a. The cut value is .500

Multiple Logistic Regression for all the parameters included in the model

Including all the variables in the model we get a classification accuracy of 86.4%, where the younger group

Variables in the Equation

		B	S.E.	Wald	df	Sig.	Exp(B)
Step 1[a]	Zscore(fractalWidth)	1.707	.709	5.799	1	.016	5.510
	Zscore(fractalMax)	.040	.931	.002	1	.966	1.040
	Zscore(Det)	-2.924	1.320	4.907	1	.027	.054
	Zscore(MaxLine)	1.427	1.930	.546	1	.460	4.164
	Zscore(MeanLine)	.526	.694	.573	1	.449	1.691
	Zscore(Ent)	2.699	2.352	1.317	1	.251	14.869
	Zscore(Lam)	-.388	1.515	.066	1	.798	.678
	Zscore(TT)	2.014	2.108	.912	1	.339	7.491
	Constant	.887	.653	1.847	1	.174	2.429

a. Variable(s) entered on step 1: Zscore(fractalWidth), Zscore(fractalMax), Zscore(Det), Zscore (MaxLine), Zscore(MeanLine), Zscore(Ent), Zscore(Lam), Zscore(TT).

Classification Table[a]

			Predicted		
			Age		Percentage Correct
Observed			.00	1.00	
Step 1	Age	.00	26	3	89.7
		1.00	5	25	83.3
	Overall Percentage				86.4

a. The cut value is .500
